# 12-year follow-up of the first endothelial keratoplasty without Descemet stripping in a 3-month newborn with Congenital Hereditary Endothelial Dystrophy (CHED)

**DOI:** 10.1186/s12886-023-03180-0

**Published:** 2023-10-25

**Authors:** Carlo Bellucci, Paolo Mora, Salvatore A. Tedesco, Stefano Gandolfi, Chiara Chierego, Roberto Bellucci

**Affiliations:** 1 Ophthalmology Unit, University Hospital of Parma, via Gramsci 14, Parma, 43126 Italy; 2 University Hospital of Verona, Verona, Italy

**Keywords:** Congenital hereditary endothelial dystrophy, Endothelial keratoplasty, Long-term follow-up, Pediatric surgery

## Abstract

**Background:**

Endothelial Keratoplasty (EK) is now considered as the standard treatment for Congenital Hereditary Endothelial Dystrophy (CHED) by many surgeons. We present the 12-year clinical outcome of the youngest operated patient with CHED in which we successfully performed a bilateral EK procedure without removing the recipient endothelium-Descemet complex.

**Case presentation:**

In November 2010 we performed EK without Descemet Stripping in a 3-month female newborn, thinking that the lower manipulation obtained by leaving the recipient endothelium–Descemet complex could be the key factor for the success of our surgery. Such a particular technique was new in newborns. The surgery was a success, but the long-term visual result was not predictable at that time. We followed the patient at 4 months, and then yearly. At the latest visit in October 2022 the visual, cognitive, and motorial developments were normal, with Best-corrected Distance Visual Acuity of 0.4 LogMAR with − 0.75 D sf + 2.75 D cyl @ 105° in the right eye (RE) and 0.4 LogMAR with + 1.50 D sf + 2.50 D cyl @ 60° in the left eye (LE). The endothelial microscope showed an unexpected healthy endothelium, with a cell count of 2383 cells/mm^2^ in the RE and of 2547 cells/mm^2^ in the LE from a starting donor count of 2900 cells/mm^2^. No secondary procedures were performed during the 12-year follow-up.

**Conclusion:**

EK without Descemet stripping has proved to be a successful procedure over time in our newborn. The unexpected healthy endothelium suggests a role of the Descemet membrane in CHED.

## Background

Congenital hereditary endothelial dystrophy (CHED) is a rare bilateral and symmetrical condition present at birth characterized by a severe endothelial cell dysfunction which leads to diffuse corneal edema. The affected corneas appear cloudy, blue-gray in color, and thick, which in turn leads to substantial impairment of visual development [1].

The older classification identified 2 types of CHED: CHED I, milder and dominantly inherited, and the more common CHED II, with a worse prognosis and recessive inheritance. The recent advances in genetic testing and the recent microscopic findings allowed for a better identification of the pathology [2], which led to the inclusion of CHED I in the spectrum of the Posterior Polymorphous Corneal Dystrophy, and CHED II - now simply called CHED – is today considered the only variant of this disease [3].

When the corneas are opaque at birth in eyes with CHED, prompt surgery is required to allow a correct development of vision. Penetrating Keratoplasty (PK) has been the procedure of choice for decades, with good results especially in older children [4–7]. Endothelial Keratoplasty (EK) without transplanting the corneal stroma has more recently shown good and promising results even in newborns [8–10].

In 2011 we reported the case of a 3-month female newborn with CHED in which we successfully performed a bilateral EK procedure without removing the recipient endothelium-Descemet complex. This procedure had never been performed in such a young patient [8]. At that time we stated that it was much too early to foresee the long-term visual result of such technique, and for this reason we want to share the 12-year clinical outcome of that patient.

## Case presentation

In November 2010 a 3-month-old female baby with CHED was referred to our attention for surgical evaluation. The young patient presented with cloudy corneas and important horizontal nystagmus. There were no signs of inflammation, photophobia, or epiphora and intraocular pressure (IOP, rebound tonometer) was 10 mmHg in the right eye (RE) and 11 mmHg in the left eye (LE). Corneal diameters were 11.5 mm (horizontal) and 11 mm (vertical) in both eyes, and corneal thickness was 680 mm in the RE and 660 mm in the LE. After discussing the condition with her parents and with the referring physician, we decided to perform Descemet-Stripping Automated Endothelial Keratoplasty (DSAEK) first, being ready to convert to PK intraoperatively in case of failure. Since at that time no report of successful DSAEK in newborns with CHED was available in literature [11], we decided not to remove the recipient endothelial-Descemet complex before implanting the donor lamella.

The LE was operated on first, and the donor lamella was successfully implanted under air. In the postoperative the recipient cornea cleared up over 2 weeks, the baby began staring at her mother while breastfeeding, and nystagmus decreased. The RE was treated after a 4-week delay, following the same surgical protocol.

Four months after surgery, nystagmus had reduced. Both corneas were transparent, with the donor lamella centered in the LE and slightly decentered temporally in the RE. The baby showed a massive improvement in her interaction with the environment, staring at lights and following the parents’ movements.

One year after surgery the corneal transparency and the position of the grafts had not changed. The baby walked into the visiting room displaying that she was able to orient well in the space. The parents confirmed that her motor and verbal development were normal and that she was able to play and interact with objects and people nearby.

Later on we made the decision to observe the corneal transparency with the slit lamp yearly, and to avoid any more detailed examinations that would have required general anesthesia, unless necessary. Despite the persisting nystagmus, the corneal reflexes remained centred on the pupil. We started measuring visual acuity at 4, finding 0.7 LogMAR uncorrected in both eyes with the employed child chart.

At the age of six the best-corrected distance visual acuity (BCVA) was 0.6 LogMAR in both eyes, with + 2.0 D cyl @ 105° in the RE and + 2.0 D cyl @ 60° in the LE. The IOP was 12 mmHg and 14 mmHg respectively. The eyes were aligned, although with poor stereopsis. The horizontal nystagmus was still present. The child started elementary school with no relevant vision problems. In 2019 at the age of 8, BCVA raised to 0.4 LogMAR in both eyes with the same cylinder correction. The COVID-19 pandemic paused the annual examinations.

**Fig. 1:**
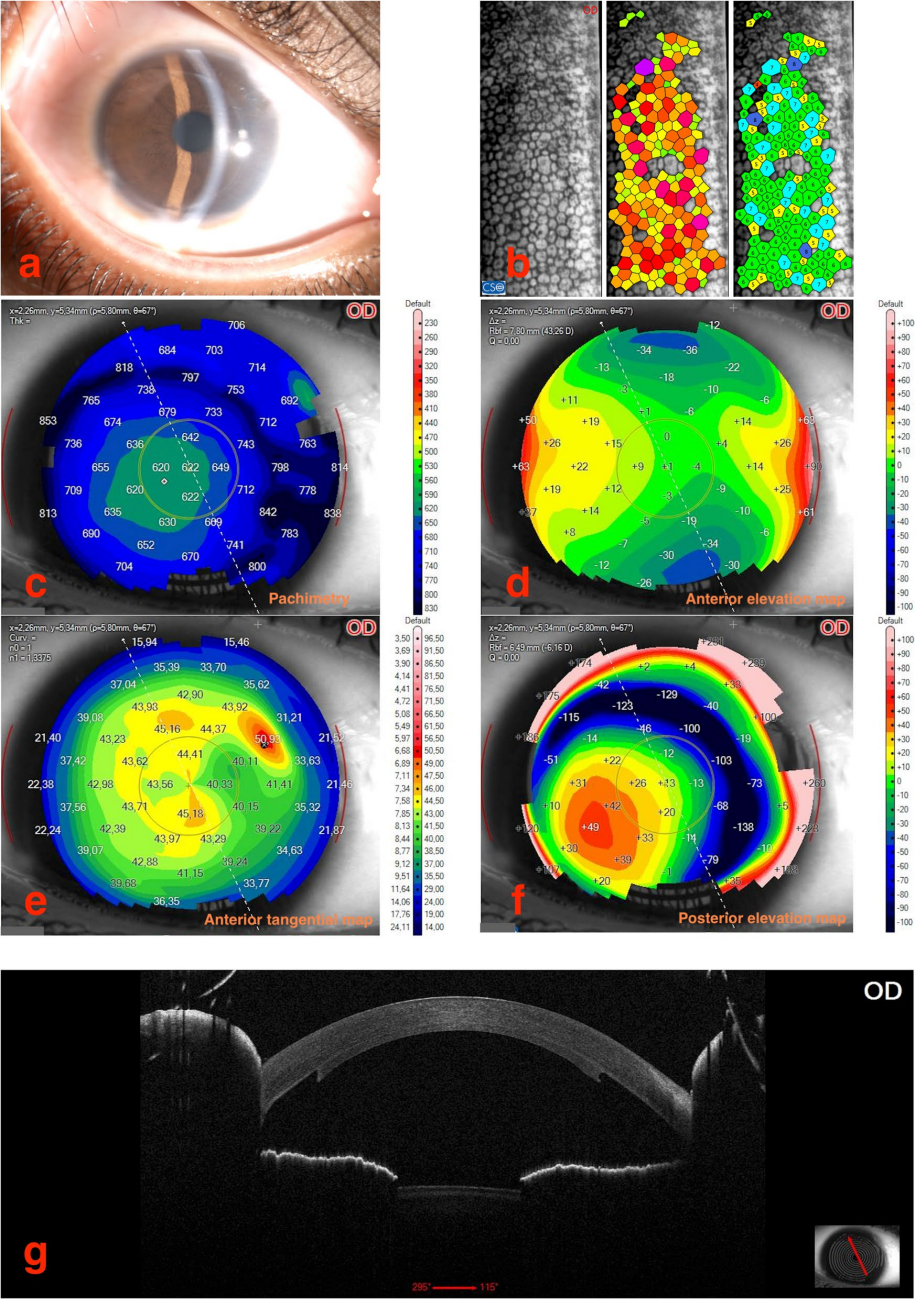
**a**-**g** Composite of the clinical and instrumental findings of the right eye of the patient at the last follow up visit in October 2022 (12 years after surgery). **a** anterior segment photography at the slit lamp examination; **b** endothelial cell count; **c** corneal pachymetry; **d**-**f** different corneal tomography maps; **g** anterior segment OCT

**Fig. 2:**
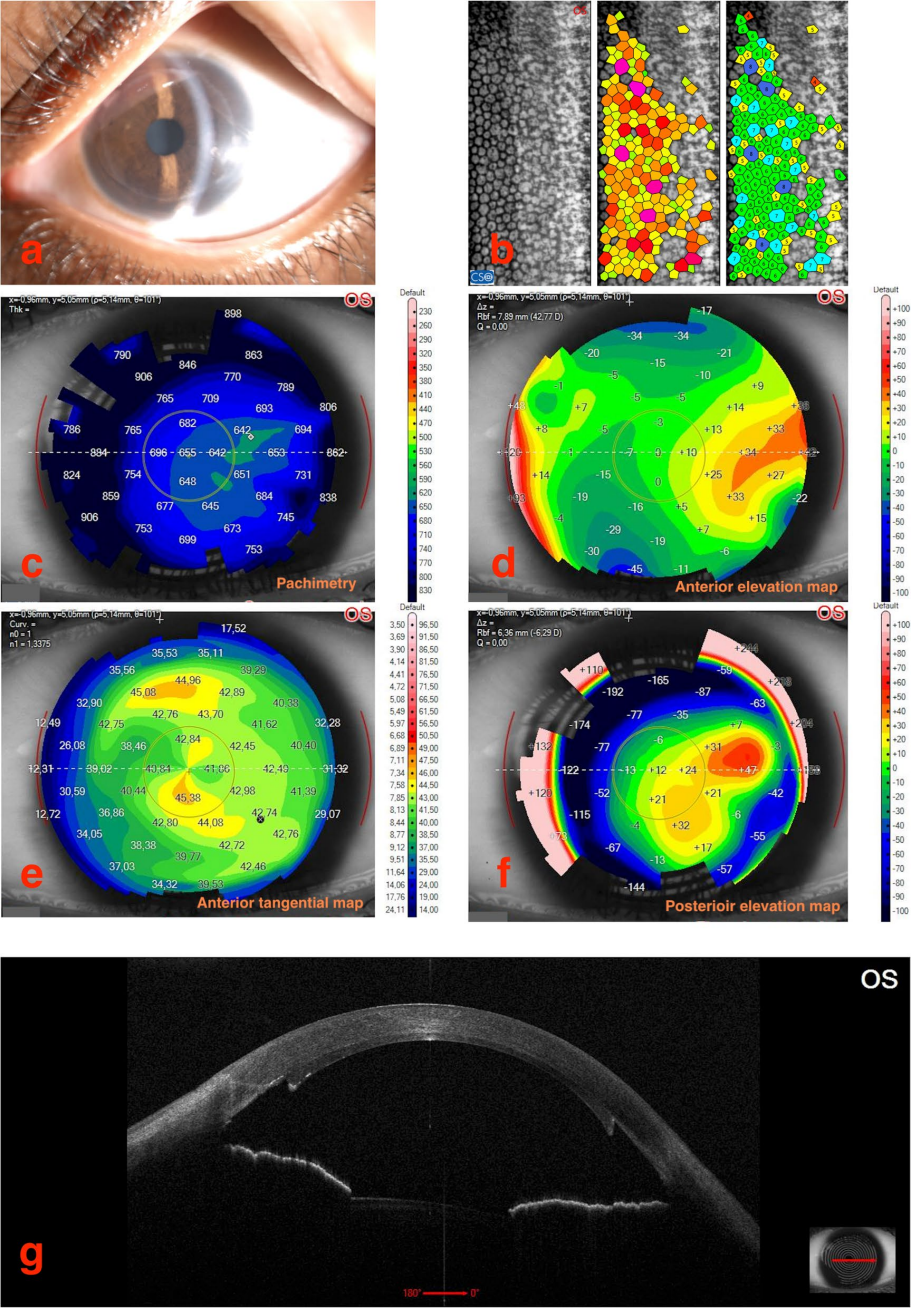
**a**-**g** Composite of the clinical and instrumental findings of the left eye of the patient at the last follow up visit in October 2022 (12 years after surgery). **a** anterior segment photography at the the slit lamp examination; **b** endothelial cell count; **c** corneal pachymetry; **d**-**f** different corneal tomography maps; **g** anterior segment OCT

The most recent visit of this patient occurred in October 2022, twelve years after surgery. Both corneas were clear and transparent. The RE lamella was slightly decentred temporally (Fig. 1a), while the LE lamella was centred (Fig. 2a). The anterior optical coherence tomography (OCT) well delineated the position of the donor lamella (Figs. 1g and 2g). The Corneal tomography showed no irregularity (Figs. 1c-f and 2c-f). The corneal endothelium was unexpectedly healthy. Cell count was 2383 cells/mm^2^ in the RE (Fig. 1b), and was 2547 cells/mm^2^ in the LE (Fig. 2b), comparing favourably with the 2900 cells/mm^2^ originally reported by the Eye Bank in both donor lamellae. BCVA was 0.4 LogMAR with − 0.75 D sf + 2.75 D cyl @ 105° in the RE and 0.4 LogMAR with + 1.50 D sf + 2.50 D cyl @ 60° in the LE. The IOP was 14 mmHg in both eyes. The eyes were aligned, the nystagmus was still present, however it was reduced as compared with that recorded before surgery. The visual function has allowed normal psychological and educational development with the girl attending the middle school without significant cognitive or motorial defects.

## Discussion and conclusions

To our knowledge, our 3-month newborn remains the youngest patient in whom a EK procedure without Descemet stripping has been performed for CHED [8]. The surgical procedure was successful, but the long-term anatomical and visual outcome of this technique was not predictable. Today we can confirm the long-term success of our choice. After 12 years both corneas are clear and transparent and the ocular anatomy is not impaired, apart from the presence of the donor lamella. No secondary procedure has been performed. Despite the persisting nystagmus the visual function allowed normal psychological and educational development. The unexpected very high endothelial cell count obtained 12 years after the graft implantation may be due to the quality of surgery, but it also suggests a possible role of the Descemet’s membrane in the pathogenesis of CHED.

Since 2011 many surgeons have performed EK in CHED with optimal results in terms of visual acuity. Concurrently along with us, Busin et al. [9] reported the same surgical procedure was performed in infants with success, but their youngest patient was a 6-month-old. At present, surgeons may choose to keep or to remove the recipient endothelial-Descemet complex, as both procedures showed comparable refractive and visual results [12, 13].

In a recent systematic review Mohebbi et al. [14] included several studies with long-term follow up after the DSAEK procedure in CHED, reporting a considerable improvement in visual acuity from the preoperative mean LogMAR of 1.36 ± 0.70 (0.49 to 3.00) to the postoperative mean LogMAR of 0.51 ± 0.33 (0.04 to 2.00). The long-term visual result of our case is in line with this finding, but the 12-year follow-up we report here is the longest to date as compared with the 8.5-year we found in the literature.

EK with or without endothelial-Descemet stripping is proposed today as the procedure of choice for the surgical treatment of CHED by many authors [14–17]. More recently Descemet membrane endothelial keratoplasty (DMEK) has been also proposed as surgical treatment option for CHED with promising results, but the steeper learning curve and the intra-operative and postoperative technical challenges of DMEK are still limiting its wide adoption in children [15, 18, 19].

We believe that PK should now be considered as a second line treatment in children with CHED, with EK as the first line surgical option. PK in children is challenging because of the low scleral rigidity, the risk for suprachoroidal hemorrhage, the difficulties in suture handling and removal, the need for prolonged steroid treatments, the high postoperative astigmatism and the high rate of graft failure and rejection [14]. In contrast EK requires shorter surgical time, has less risk of the serious complications mentioned above, is associated with shorter recovery time, and provides not an inferior long term visual outcome [16].

Our patient did not develop amblyopia or graft rejection, did not require any secondary procedures, and is living a normal life. We cannot predict how long the donor endothelium will remain functional, but any future surgery will take place in a young adult or in an adult patient without the technical difficulties that are typical of newborns.

## Data Availability

All data and material are available from the corresponding author.

## Abbreviations

EK: Endothelial Keratoplasty
CHED: Congenital Hereditary Endothelial Dystrophy
RE: Right Eye
LE: Left Eye
PK: Penetrating Keratoplasty
DSAEK: Descemet-Stripping Automated Endothelial Keratoplasty
BCVA: Best-corrected Distance Visual Acuity
IOP: Intraocular Pressure
OCT: Optical Coherence Tomography
DMEK: Descemet Membrane Endothelial Keratoplasty

## References

[1] Vanathi M, Raj N, Kusumesh R, Aron N, Gupta N, Tandon R (2022). Update on pediatric corneal diseases and keratoplasty. Surv Ophthalmol.

[2] Aldave AJ, Han J, Frausto RF (2013). Genetics of the corneal endothelial dystrophies: an evidence-based review. Clin Genet.

[3] Nischal KK (2015). Genetics of congenital corneal opacification - impact on diagnosis and treatment. Cornea.

[4] Sajjadi H, Javadi MA, Hemmati R (1995). Results of penetrating keratoplasty in CHED. Congenital hereditary endothelial dystrophy. Cornea.

[5] Al-Rajhi AA, Wagoner MD (1997). Penetrating keratoplasty in congenital hereditary endothelial dystrophy. Ophthalmology.

[6] Schaumberg DA, Moyes AL, Gomes JAP (1999). Corneal transplantation in young children with congenital hereditary endothelial dystrophy. Multicenter Pediatric keratoplasty study. Am J Ophthalmol.

[7] Javadi MA, Baradaran-Rafii AR, Zamani M (2003). Penetrating keratoplasty in young children with congenital hereditary endothelial dystrophy. Cornea.

[8] Bellucci R, Chierego C, Bellucci C (2011). Endothelial keratoplasty in a newborn baby with CHED. Cornea.

[9] Busin M, Beltz J, Scorcia V (2011). Descemet-stripping automated endothelial keratoplasty for congenital hereditary endothelial dystrophy. Arch Ophthalmol.

[10] Goshe JM, Li JY, Terry MA (2012). Successful Descemet’s stripping automated endothelial keratoplasty for congenital hereditary endothelial dystrophy in a pediatric patient. Int Ophthalmol.

[11] Pineda RII, Jain V, Shome D (2010). Descemet’s stripping endothelial keratoplasty: is it an option for congenital hereditary endothelial dystrophy?. Int Ophthalmol.

[12] Ashar JN, Ramappa M, Chaurasia S (2013). Endothelial keratoplasty without descemet’s stripping in congenital hereditary endothelial dystrophy. J AAPOS.

[13] Asif MI, Bafna RK, Sharma N, Kaginalkar A, Sinha R, Agarwal T (2021). Microscope integrated optical coherence tomography guided Descemet stripping automated endothelial keratoplasty in congenital hereditary endothelial dystrophy. Clin Ophthalmol.

[14] Mohebbi M, Mehrpour M, Sanij AD, Mohammadi N, Mirghorbani M (2022). Pediatric endothelial keratoplasty: a systematic review and individual participant data meta-analysis. Graefes Arch Clin Exp Ophthalmol.

[15] Mandal S, Asif MI, Maharana PK, Sharma N, Titiyal JS (2022). A review of techniques and outcomes of endothelial keratoplasty in congenital hereditary endothelial dystrophy. Indian J Ophthalmol.

[16] Al-Dahan D, AlRajhi A, AlHazzani A, Alabdulwahid R, Alqarni A, Ahad MA (2022). Penetrating keratoplasty versus descemet stripping automated endothelial keratoplasty in children with congenital hereditary endothelial dystrophy: long-term results. Eye Contact Lens.

[17] Ashar JN, Ramappa M, Vaddavalli PK (2013). Paired-eye comparison of descemet’s stripping endothelial keratoplasty and penetrating keratoplasty in children with congenital hereditary endothelial dystrophy. Br J Ophthalmol.

[18] Wu F, Oatts JT, Schallhorn JM (2021). Bilateral Descemet membrane endothelial keratoplasty in an infant with congenital hereditary endothelial dystrophy. Cornea.

[19] Pereira NC, Pereira Gomes JÁ, Tonin C, Verardo FO, Felippe RS (2021). Dos Santos Forseto A. Descemet membrane endothelial keratoplasty in children. Cornea.

